# Unraveling the psychological burden of Behçet disease: the impact of anxiety and depression on health-related quality of life outcomes

**DOI:** 10.1186/s13023-026-04433-z

**Published:** 2026-07-07

**Authors:** Samar Tharwat, Mohamad Nassan, Nasim Jaber, Mo’min Khatib, Hamza Aljubaeh, Omar Abumunshar

**Affiliations:** 1 Rheumatology & Immunology Unit, Department of Internal Medicine, Faculty of Medicine, Mansoura University, Mansoura, Egypt; 2 Department of Internal Medicine, Faculty of Medicine, Horus University, New Damietta, Egypt; 3 Faculty of Medicine, Mansoura University, Mansoura, Egypt; 4 Mansoura University Hospital, El Gomhouria St, Mansoura, Dakahlia Governorate 35511 Egypt

**Keywords:** Behçet’s disease, Anxiety, Depression, Health-related quality of life, HADS, SF-36, Multidisciplinary care

## Abstract

**Background:**

Behçet’s disease (BD) is a chronic, inflammatory multisystem disease that has impact both physical health and mental well-being. However, the psychological impact of BD remains underexplored, particularly in Arabic-speaking populations. The aim of this study was to assess the prevalence of anxiety and depression in Arabic-speaking BD patients, evaluate their association with health-related quality of life (HRQoL), and identify key predictors of psychological distress in these patients.

**Methods:**

This cross-sectional study included 192 BD patients recruited from our rheumatology clinic and an online survey among BD patients. Psychological distress was assessed using the Arabic version of Hospital Anxiety and Depression Scale (HADS), while HRQoL was assessed via the Arabic version of Short Form 36 (SF-36) Health Survey. Univariate and multivariate regression analyses were performed to identify main factors linked with anxiety and depression.

**Results:**

Anxiety and depression were highly prevalent in BD patients, with 53.1% of the study participants exhibiting abnormal anxiety levels and 38.5% showing abnormal depression scores. Higher levels of psychological distress were statistically significantly associated with BD disease activity and elevated erythrocyte sedimentation rate levels (*p* = 0.017). Additionally, patients with greater anxiety and depression scores had significantly lower HRQoL scores across all SF-36 domains (*p* < 0.001). Gender differences were observed, with females showing higher anxiety rates (*p* = 0.006).

**Conclusions:**

Anxiety and depression are major contributors to reduced HRQoL in BD patients, necessitating a multidisciplinary approach that integrates mental health care into disease management.

**Clinical trial number:**

Not applicable.

## Introduction

Behçet’s disease (BD) is a chronic, multisystem inflammatory disease that usually manifests with recurrent oral and genital ulcers, and uveitis [1]. BD can affect multiple organ systems, including the ocular, integumentary, musculoskeletal, gastrointestinal, and central neurological systems [2]. Although its exact cause is uncertain, BD is believed to result from an aberrant immunological response elicited by external factors in genetically predisposed individuals [3]. Managing BD requires a thorough, multidisciplinary approach. This approach aims to reduce inflammation and prevent the worsening of the disease. It uses various treatments, including corticosteroids, immunosuppressive drugs, and biologic therapies [4].

Patients with BD frequently experience significant psychological distress, with anxiety and depression being among the most commonly reported mental health disorders in this population [5]. The chronic, unpredictable nature of BD, coupled with its painful and disabling manifestations, contributes to an increased risk of emotional distress and psychiatric comorbidities [6]. In addition, inflammation can contribute to the development of mood disorder, with cytokines involved in BD pathogenesis, including tumor necrosis factor-alpha (TNF-alpha) and several interleukins, being linked with neurobiological processes pertinent to depression and anxiety [1, 7].

Health-related quality of life (HRQoL), a multifaceted concept that captures the effects of illness on physical, emotional, and social wellbeing, extending beyond clinical indicators, is of paramount importance in the management of chronic diseases [8]. Within the context of BD, HRQoL is diminished due to the disease’s relapsing course [9], the presence of persistent pain and fatigue, and the influence of systemic involvement on everyday activities [10]. Physical limitations such as joint pain, visual impairment, and neurological symptoms play a significant role in contributing to disability, but other factors such as emotional distress in terms of anxiety, depression, and uncertainty about disease progression also impair HRQoL [5].

Although several studies [11–13] from Turkey, Iran, and European countries have explored anxiety, depression, and QoL in BD, important gaps remain. Most available data originate from non-Arab populations and may not fully capture the sociocultural context of Arabic-speaking patients, where stigma surrounding mental health, differences in healthcare access, and family-centered coping mechanisms may substantially influence psychological outcomes. Therefore, data specifically addressing the psychological burden of BD in Arabic-speaking populations using culturally validated instruments remain scarce. In addition, universally acknowledged and validated tools, such as the Hospital Anxiety and Depression Scale (HADS) and the SF-36 Health Survey, are underutilized in BD research.

Based on these gaps, we hypothesized that anxiety and depression are prevalent among Arabic-speaking patients with BD and are significantly associated with impaired HRQoL We further hypothesized that psychological distress is influenced by disease activity, inflammatory markers, and key demographic and clinical factors.

Therefore, this study aims to investigate the prevalence of anxiety and depression among Arabic-speaking BD patients, to assess their impact on HRQoL, and to identify key predictors of psychological distress.

## Methods

### Study design and setting

This cross-sectional study included 192 patients diagnosed with BD. Participants were recruited through two sources: attendees at our institution’s BD clinic, and respondents to an online survey shared through social networks and patient advocacy groups. Data were collected entirely through a standardized online, self-administered survey developed using Google Forms. This approach was uniformly applied to all participants, including those recruited from the rheumatology clinic, who were invited to complete the same online questionnaire, as well as those recruited via social media and patient support groups.

Eligible participants were adult patients diagnosed with BD according to the International Study Group (ISG) criteria for BD [14], with confirmation from an expert rheumatologist as documented in their medical records. For online participants, diagnostic confirmation was ensured by: (i) requiring self-reported prior rheumatologist-confirmed diagnosis; (ii) retrospective verification of ISG criteria through detailed clinical questions. Subjects with a history of malignancies and any other chronic rheumatic, musculoskeletal, neurological, and psychiatric disease preceding the development of BD were not included in the study. The recruitment process spanned from March 2024 to January 2025.

#### Sample size calculation

Sample size determination was performed using G*Power software to ascertain sufficient statistical power for identifying meaningful relationships among anxiety, depression, and HRQoL within the BD patients. Employing a medium effect size (f² = 0.15), a significance threshold (α) of 0.05, and a power of 80%, the analysis revealed that a sample of 92 participants was necessary for a multiple regression model incorporating five predictors. To account for potential dropouts or incomplete data, the sample size was increased by 20%, resulting in a final target of 110 participants.

#### Ethical consideration

This study was conducted in compliance with the the Declaration of Helsinki and approved by the Institutional Research Board of the Faculty of Medicine at Mansoura University (approval no: R.25.02.3037). Participants’ collection was facilitated through an electronic survey, with all participants providing electronic consent beforehand. All participants received a full description of the purpose of the study, and confidentiality measures. The website through which the survey took place was carefully designed to maintain both the security and privacy of information, with access granted only to approved researchers. Participants did not receive any form of financial or material compensation for their involvement in the study.

#### Baseline demographic characteristics

Demographic data included age, gender, marriage status, residence (urban and rural), educational level, and work status. Socioeconomic level was grouped into three categories: low, moderate, and high, and determined through participants’ self-described financial status. In addition, life habits, such as cigarette smoking, consumption of alcohol, and exercise, were documented.

#### Clinical, laboratory and therapeutic data

The survey included questions about medical background. Symptoms including recurrent oral and genital ulcers, ocular manifestations, vasculitis, and neurological symptoms were documented. In addition, information about both ongoing and past therapies, including colchicine, corticosteroids, immunosuppressive drugs, and biologic drugs, was obtained.

Laboratory data were collected from patients’ medical records, including complete blood count, erythrocyte sedimentation rate, in addition to liver enzymes. Patients were asked to provide their most recent laboratory test results related to these parameters. Laboratory data were categorized based on standard reference ranges.

Disease activity was obtained using patient self-report and categorized as none, mild, moderate, or severe. This approach was chosen due to the cross-sectional nature of the study and the inclusion of online participants, for whom contemporaneous physician-based assessment was not feasible. Self-reported disease activity reflects patients’ perceived disease burden, which is particularly relevant when assessing psychological distress and HRQoL.

#### Hospital Anxiety and Depression Scale (HADS)

The HADS was utilized to assess anxiety and depression severity in participants in the current study. HADS is a widely utilized self-rated screening tool designed to detect significant anxiety and depression in both community and hospital settings. HADS consists of 14 items, seven for anxiety (HADS-A) and seven for depression (HADS-D) with each rated with a four-point Likert scale. In the current study, the validated Arabic version of HADS was used to ensure its suitability for use with Arabic-speaking participants [15]. The Arabic version of the HADS is a reliable and valid tool. Its reported Cronbach’s alpha values are 0.83 for the anxiety subscale and 0.77 for the depression subscale, confirming its reliability for use in Arabic-speaking populations [15].

#### Short form 36 (SF-36)

The SF-36 Health Survey is a well-known and widely used tool for evaluation of HRQoL. It covers eight key domains: physical functioning, role limitations caused by physical or emotional health issues, energy levels and fatigue, emotional well-being, social functioning, pain, and general perceptions of health. For this study, we used the Arabic version of the SF-36, which has been translated and validated in previous research [16, 17], confirming its reliability and validity within Arabic-speaking populations.

### Statistical analysis

Statistical analysis was conducted using SPSS version 26.0 (IBM Corp, Armonk, NY, USA). Continuous variables were presented as means ± standard deviations (SD) or medians with minimum and maximum based on data distribution, while categorical variables were expressed as frequencies and percentages. Normality of distribution was assessed using the Kolmogorov-Smirnov test. Analysis of Variance (ANOVA) test was used to compare parametric variables, while Kruskal–Wallis’s test was used to compare non-parametric variables between 3 subgroups of anxiety and depression. Chi-square tests were applied for categorical data. Univariate and multivariate linear regression analyses were performed to identify independent predictors of anxiety and depression. The regression results were reported as Beta coefficients with 95% confidence intervals (CI). Statistical significance was set at *p* < 0.05. Effect sizes for Kruskal-Wallis tests were calculated using eta-squared (η²) as η² = (H - k + 1) / (n - k), where H is the Kruskal-Wallis statistic, k = 3 groups, and *n* = 192. According to Cohen’s guidelines, η² values of 0.01, 0.06, and 0.14 were interpreted as small, medium, and large effects, respectively.

## Results

Table 1 describes the baseline demographics of 192 BD patients, categorized according to HADS scores for anxiety and depression. The anxiety subgroups comprised 24.5% normal, 22.4% borderline, and 53.1% abnormal cases. In contrast, the depression subgroups consisted of 30.7% normal, 30.7% borderline, and 38.5% abnormal cases. Gender difference are significant for anxiety, with more females in the abnormal group (60.8%) compared to males (39.2%) (*p* = 0.006). Regarding life habits, exercise practice differs significantly among depression subgroups (*p* = 0.048), with the lowest exercise engagement in the abnormal depression group (17.6%).

**Table 1:** Baseline demographic characteristics of the study Behçet disease patients (*n* = 192), comparing between anxiety and depression subgroups

Variable mean ± SD, *n* (%), median(min.-max.)	Total (*n* = 192)	HADS-A				HADS-D			
		Normal (*n* = 47)	Borderline (*n* = 43)	Abnormal (*n* = 102)	*P*	Normal (*n* = 59)	Borderline (*n* = 59)	Abnormal (*n* = 74)	*P*
%	100%	24.5%	22.4%	53.1%	-	30.7%	30.7%	38.5%	
Age	33.25 ± 8.98	34.60 ± 11.10	33.67 ± 8.68	32.45 ± 7.970	0.377	32.17 ± 9.72	33.53 ± 8.39	33.89 ± 8.86	0.763
Gender									
Male	94 (49.0)	25 (53.2)	29 (67.4)	40 (39.2)	**0.006**	26 (44.1)	31 (52.5)	37 (50.0)	0.638
Female	98 (51.0)	22 (46.8)	14 (32.6)	62 (60.8)		33 (55.9)	28 (47.5)	37 (50.0)	
Marital status									0.651
Not Married	90 (46.9)	22 (46.8)	21 (48.8)	47 (46.1)	0.955	25 (42.4)	30 (50.8)	35 (47.3)	
Married	102 (53.1)	25 (53.2)	22 (51.2)	55 (53.9)		34 (57.6)	29 (49.2)	39 (52.7)	
Living alone	17 (8.9)	4 (8.5)	6 (14.0)	7 (6.9)	0.388	5 (8.5)	4 (6.8)	8 (10.8)	0.713
Residence									
Rural	46 (24.0)	9 (19.1)	12 (27.9)	25 (24.5)	0.612	13 (22.0)	12 (20.3)	21 (28.4)	0.512
Urban	146 (76.0)	38 (80.9)	31 (72.1)	77 (75.5)		46 (78.0)	47 (79.7)	53 (71.6)	
Education level									
Not educated	3 (1.6)	1 (2.1)	1 (2.3)	1 (1.0)	0.785	2 (3.4)	-	1 (1.4)	0.326
Educated	189 (98.4)	46 (97.9)	42 (97.7)	101 (99.0)		57 (96.6)	59 (100.0)	73 (98.6)	
Employment status									
Not working	91(47.4)	24 (51.1)	21 (48.8)	46 (45.1)	0.777	31 (52.5)	22 (37.3)	38 (51.4)	0.173
Working	101 (52.6)	23 (48.9)	22 (51.2)	56 (54.9)		28 (47.5)	37 (62.7)	36 (48.6)	
Socioeconomic status									
Low	41 (21.4)	10 (21.3)	11 (25.6)	20 (19.6)	0.374	10 (16.9)	12 (20.3)	19 (25.7)	0.448
Moderate	150 (78.1)	37 (78.7)	31 (72.1)	82 (80.4)		48 (81.4)	47 (79.7)	55 (74.3)	
High	1 (0.5)	-	1 (2.3)	-		1 (1.7)	-	-	
Life habits									
Smoking	56 (29.2)	9 (19.1)	14 (32.6)	33 (32.4)	0.228	17 (28.8)	16 (27.1)	23 (31.1)	0.908
Alcohol consumption	3 (1.6)	-	1 (2.3)	2 (2.0)		2 (3.4)	-	1 (1.4)	0.309
Exercise practice	53 (27.6)	14 (29.8)	16 (37.2)	23 (22.5)	0.609 0.183	20 (33.9)	20 (33.9)	13 (17.6)	**0.048**
Treatment payment system									
Health insurance	77 (40.1)	19 (40.4)	14 (32.6)	44 (43.1)	0.404	20 (33.9)	28 (47.5)	29 (39.2)	0.389
State expense	42 (21.9)	6 (12.8)	11 (25.6)	25 (24.5)	0.430	6 (10.2)	17 (28.8)	19 (25.7)	0.108
Patient expense	135 (70.3)	34 (72.3)	25 (58.1)	76 (74.5)	0.135	44 (74.6)	41 (69.5)	50 (67.6)	0.670

Bold: *p* < 0.05

HADS: Hospital Anxiety and Depression Scale; HADS-A: Hospital Anxiety and Depression Scale - Anxiety Subscale; HADS-D: Hospital Anxiety and Depression Scale - Depression Subscale

Clinical, laboratory, and therapeutic data of the BD patients, categorized based on Hospital HADS, are shown in Table 2. Self-reported disease activity showed a significant association with anxiety and depression (*p* = 0.038), with more severe cases in the abnormal groups. Significant clinical manifestations associated with anxiety and depression include erythema nodosum, pseudofolliculitis, skin ulcers, recurrent fever, abdominal symptoms, psychiatric symptoms, and central nervous system involvement. Laboratory findings indicate a higher prevalence of high ESR levels in anxiety cases (*p* = 0.017).

**Table 2:** Clinical, laboratory and therapeutic data of the study Behçet disease patients (*n* = 192), comparing between anxiety and depression subgroups

Variable mean ± SD, *n* (%), median(min.-max.)	Total (*n* = 192)	HADS-A				HADS-D			
		Normal (*n* = 47)	Borderline (*n* = 43)	Abnormal (*n* = 102)	*P*	Normal (*n* = 59)	Borderline (*n* = 59)	Abnormal (*n* = 74)	*P*
Clinical data of Behcet disease									
Age at disease onset (years)	23.91 ± 8.54	24.23 ± 9.07	24.14 ± 8.65	23.66 ± 8.32	0.911	23.83 ± 8.42	22.95 ± 7.98	24.73 ± 9.08	0.491
Diagnostic delay (months)	10 (1–72)	10 (0-108)	8 (1–60)	9.5 (1–72)	0.614	6 (0-108)	12 (1–72)	8 (1–60)	0.614
Disease duration (years)	7 (0–46)	9 (0.5–35)	8 (1–46)	7 (0–30)	0.299	7 (0.5–35)	9 (0–41)	7 (0–46)	0.800
Family history of Behcet disease	22 (11.5)	5 (10.6)	1 (2.3)	16 (15.7)	0.068	5 (8.5)	8 (13.6	9 (12.2	0.667
Self-reported disease activity									
No	7 (3.6)	4 (8.5)	11 (25.6)	3 (2.9)	**0.038**	5 (8.5)	19 (32.2)	2 (2.7)	**0.038**
Mild	53 (27.6)	18 (38.3)	22 (51.2)	24 (23.5)		19 (32.2)	29 (49.2)	15 (20.3)	
Moderate	80 (41.7)	18 (38.3)	10 (23.3)	40 (39.2)		19 (32.2)	11 (18.6)	32 (43.2)	
Sever	52 (27.1)	7 (14.9)	-	35 (34.3)		16 (27.1)	-	25 (33.8)	
Cumulative manifestations of Behcet disease									
Recurrent oral aphthosis	183 (95.3)	44 (93.6)	42 (97.7)	97 (95.1)	0.352	55 (93.2)	59 (100.0)	69 (93.2)	0.142
Erythema nodosum	115 (59.9)	20 (42.6)	31 (72.1)	64 (62.7)	**0.019**	30 (50.8)	38 (64.4)	47 (63.5)	0.270
Pseudofolliculitis	102 (53.1)	10 (21.3)	28 (65.1)	64 (62.7)	**< 0.001**	21 (35.6)	35 (59.3)	46 (62.2)	**0.005**
Skin ulcers	109 (56.8)	18 (38.3)	28 (65.1)	63 (61.8)	**0.037**	25 (42.4)	35 (59.3)	49 (66.2)	**0.010**
Recurrent genital aphthosis	129 (67.2)	30 (63.8)	35 (81.4)	64 (62.7)	0.115	38 (64.4)	40 (67.8)	51 (68.9)	0.576
Recurrent attacks of fever	110 (57.3)	16 (34.0)	30 (69.8)	64 (62.7)	**0.001**	26 (44.1)	35 (59.3	49 (66.2)	**0.003**
Abdominal pain/ nausea/ diarrhoea	119 (62.0)	14 (29.8)	30 (69.8)	75 (73.5)	**< 0.001**	31 (52.5)	38 (64.4)	50 (67.6)	0.064
Endoscopy	42 (21.9)	10 (21.3)	7 (16.3)	25 (24.5)	0.536	11 (18.6)	14 (23.7)	17 (23.0)	0.689
Macroscopic/microscopic inflammatory lesions seen by Endoscopy	34 (17.7)	3 (6.4)	6 (14.0)	25 (24.5)	**0.023**	8 (13.6)	12 (20.3)	14 (18.9)	0.514
Ocular involvement	117 (60.9)	28 (59.6)	30 (69.8)	59 (57.8)	0.638	36 (61.0)	38 (64.4)	43 (58.1)	0.557
CNS manifestations	25 (13.0)	4 (8.5)	5 (11.6)	16 (15.7)	0.479	5 (8.5)	5 (8.5)	15 (20.3)	**0.042**
PNS manifestations	70 (36.5)	11 (23.4)	16 (37.2)	43 (42.2)	0.105	20 (33.9)	16 (27.1)	34 (45.9)	0.064
Psychiatric symptoms	129 (67.2)	13 (27.7)	31 (72.1)	85 (83.3)	**< 0.001**	34 (57.6)	36 (61.0)	59 (79.7)	**0.005**
Vascular involvement	102 (53.1)	22 (46.8)	24 (55.8)	56 (54.9)	0.541	27 (45.8)	31 (52.5)	44 (59.5)	0.115
Cardiac involvement	45 (23.4)	9 (19.1)	5 (11.6)	31 (30.4)	**0.033**	15 (25.4)	10 (16.9)	20 (27.0)	0.222
Arrhythmia	70 (36.5)	15 (31.9)	14 (32.6)	41 (40.2)	0.466	23 (39.0)	17 (28.8)	30 (40.5)	0.302
Myopericarditis	11 (5.7)	1 (2.1)	2 (4.7)	8 (7.8)	0.349	8 (13.6)	1 (1.7)	2 (2.7)	0.**011**
Intracardiac thrombosis	10 (5.2)	2 (4.3)	1 (2.3)	7 (6.9)	0.510	4 (6.8)	2 (3.4)	4 (5.4)	0.711
Lymphadenopathy	30 (15.6)	4 (8.5)	7 (16.3)	19 (18.6)	0.285	10 (16.9)	11 (18.6)	9 (12.2)	0.666
Cardiomyopathy	4 (2.1)	1 (2.1)	-	3 (2.9)	0.531	2 (3.4)		2 (2.7)	0.387
Pleuritis	23 (12.0)	3 (6.4)	5 (11.6)	15 (14.7	0.351	8 (13.6)	6 (10.2)	9 (12.2)	0.857
Orchitis	20 (10.4)	1 (2.1)	5 (11.6)	14 (13.7	0.102	6 (10.2)	7 (11.9)	7 (9.5)	0.938
Associated comorbidities									
Peripheral vascular disease	22 (11.5)	3 (6.4)	5 (11.6)	14 (13.7)	0.416	4 (6.8)	7 (11.9)	11 (14.9)	0.216
IHD	9 (4.7)	3 (6.4)	2 (4.7)	4 (3.9)	0.806	4 (6.8)	1 (1.7)	4 (5.4)	0.445
Cerebrovascular stroke	13 (6.8)	3 (6.4)	4 (9.3)	6 (5.9)	0.750		6 (10.2)	7 (9.5)	**0.045**
Systemic hypertension	38 (19.8)	9 (19.1)	8 (18.6)	21 (20.6)	0.885	8 (13.6)	11 (18.6)	19 (25.7)	0.171
Diabetes millatus	21 (10.9)	3 (6.4)	5 (11.6)	13 (12.7)	0.529	2 (3.4)	12 (20.3)	7 (9.5)	**0.014**
Dyslidemia	42 (21.9)	6 (12.8)	8 (18.6)	28 (27.5)	0.120	9 (15.3)	17 (28.8)	16 (21.6)	0.177
Hypothyroidism	18 (9.4)	1 (2.1)	7 (16.3)	10 (9.8)	0.085	5 (8.5)	9 (15.3)	4 (5.4)	0.150
Heart failure	5 (2.6)	1 (2.1)	1 (2.3)	3 (2.9)	0.933	3 (5.1)	1 (1.7)	1 (1.4)	0.456
Sever infection	35 (18.2)	2 (4.3)	7 (16.3)	26 (25.5)	**0.004**	12 (20.3	8 (13.6)	15 (20.3	0.515
Malignancy	1 (0.5)	-	-	1 (1.0)	0.627	-	-	1(1.4)	0.406
Laboratory data									
Abnormal WBCs	57 (29.7)	13 (27.7)	9 (20.9)	35 (34.3)	0.257	18 (30.5)	20 (33.9)	19 (25.7)	0.580
Anemia	27 (14.1)	3 (6.4)	5 (11.6)	19 (18.6)	0.119	8 (13.6)	11 (18.6)	8 (10.8)	0.431
Thrombocytopenia	16 (8.3)	2 (4.3)	3 (7.0)	11 (10.8)	0.381	4 (6.8)	8 (13.6)	4 (5.4)	0.209
Elevated liver enzymes	16 (8.3)	5 (10.6)	3 (7.0)	8 (7.8)	0.794	2 (3.4)	7 (11.9)	7 (9.5)	0.226
High ESR	33 (17.2)	4 (8.5)	4 (9.3)	25 (24.5)	**0.017**	5 (8.5)	10 (16.9)	18 (24.3)	**0.055**
BD therapeutic data									
Colchicine	163 (84.9)	43 (91.5)	40 (93.0)	86 (84.3)	0.358	45 (76.3)	55 (93.2)	63 (85.1)	**0.016**
Corticosteroids	154 (80.2)	33 (70.2)	43 (100.0)	83 (81.4)	0.212	48 (81.4)	48 (81.4)	58 (78.4)	0.828
Antimalarials	13 (6.8)	2 (4.3)	3 (7.0)	8 (7.8)	0.719	4 (6.8)	4 (6.8)	5 (6.8)	1.000
Azathioprine	59 (30.7)	15 (31.9)	15 (34.9)	29 (28.4)	0.860	19 (32.2)	21 (35.6)	19 (25.7)	0.443
Sulfasalazine	7 (3.6)	3 (6.4)		4 (3.9)	0.238	2 (3.4)	1 (1.7)	4 (5.4)	0.512
Methotrexate	15 (7.8)	3 (6.4)	5 (11.6)	7 (6.9)	0.702	7 (11.9)	4 (6.8)	4 (5.4)	0.346
Mycophenolate	8 (4.2)	2 (4.3)	2 (4.7)	4 (3.9)	0.999	2 (3.4)	3 (5.1)	3 (4.1)	0.907
Cyclosporine	13 (6.8)	7 (14.9)	3 (7.0)	3 (2.9)	**0.028**	4 (6.8)	7 (11.9)	2 (2.7)	0.110
Adalimumab	16 (8.3)	6 (12.8)	3 (7.0)	7 (6.9)	0.449	4 (6.8)	7 (11.9)	5 (6.8)	0.526
Infliximab	12 (6.3)	3 (6.4)	4 (9.3)	5 (4.9)	0.712	5 (8.5)	5 (8.5)	2 (2.7)	0.260
Rituximab	4 (2.1)	1 (2.1)	1 (2.3)	2 (2.0)	0.998	1 (1.7)	2 (3.4)	1 (1.4)	0.693

Bold: *p* < 0.05

CNS: central nervous system, ESR: erythrocyte sedimentation rate, IHD: ischemic heart disease, PNS: peripheral nervous system, WBCs: white blood cells

Table 3 presents univariate and multivariate linear regression analysis examining factors associated with HADS-anxiety (HADS-A) and HADS-depression (HADS-D) in BD patients. For HADS-A, severe disease activity (*p* = 0.042), abdominal symptoms (*p* = 0.004), and high ESR (*p* = 0.016) were significantly associated in the multivariate model. Interestingly, cyclosporine use was negatively associated with anxiety (*p* = 0.001). For HADS-D, psychiatric symptoms were the strongest predictor (*p* < 0.001), while recurrent fever and severe disease activity were significant in the univariate model but lost significance in multivariate analysis.

**Table 3:** Univariate and multivariate linear regression analysis of HADS-anxiety and HADS-depression

Variable	HADS-A						HADS-D						
	Univariate analysis		Multivariate analysis				Variable	Univariate analysis		Multivariate analysis			
	Beta	*P*value	Beta	*P *value	Confidence interval			Beta	*P *value	Beta	*P *value	Confidence interval	
					Lower bound	Higher bound						Lower bound	Higher bound
Constant				0.000	4.759	9.525					0.000	2.834	7.053
Gender	-0.138	**0.056**	-0.125	**0.071**	-2.646	0.109	Exercise practice	0.127	0.078	-0.128	0.100	-2.532	0.224
Sever disease activity	0.267	**< 0.001**	0.147	0.042	0.061	3.390	Sever disease activity	0.177	**0.014**	0.126	0.127	-0.331	2.643
Erythema nodosum	0.168	**0.022**	0.049	0.521	-1.037	2.037	Pseudofolliculitis	0.247	**0.001**	0.074	0.380	-0.737	1.919
Pseudofolliculitis	0.303	**< 0.001**	0.023	0.753	-1.249	1.724	Skin ulcers	0.268	**< 0.001**	0.104	0.233	-0.549	2.238
Recurrent attacks of fever	0.279	**< 0.001**	0.026	0.731	-1.292	1.836	Recurrent attacks of fever	0.268	**< 0.001**	0.136	0.102	-0.226	2.464
Abdominal pain/ nausea/ diarrhoea	0.382	**< 0.001**	0.230	0.004	0.799	4.160	CNS manifestations	0.179	**0.021**	0.168	0.067	-0.133	3.926
Macroscopic/microscopic inflammatory lesions seen by Endoscopy	0.229	**0.003**	0.064	0.370	-0.982	2.616	Psychiatric symptoms	0.295	**< 0.001**	0.234	**0.006**	0.625	3.555
Psychiatric symptoms	0.511	**< 0.001**	0.312	**< 0.001**	1.871	5.125	Myopericarditis	-0.093	0.240	-0.046	0.553	-3.688	1.985
Cardiac involvement	0.204	**0.009**	0.093	0.180	-0.527	2.785	Cerebrovascular stroke	0.110	0.130	0.045	0.627	-2.080	3.438
Sever infection	0.267	**0.001**	0.040	0.566	-1.231	2.240	Diabetes millatus	0.135	0.085	-0.020	0.800	-2.238	1.729
High ESR	0.269	**< 0.001**	0.167	**0.016**	0.418	4.003	High ESR	0.135	0.085	0.143	0.069	− 0.120	3.120
Cyclosporine	0.213	**0.009**	-0.235	**0.001**	-6.695	-1.833	Colchicine	0.091	0.222	0.071	0.380	-1.121	2.922

Bold: *p* < 0.05

Tables 4 and 5 show HRQoL data in the study BD patients, comparing subgroups based on HADS-A and HADS-D scores. Patients with higher anxiety and depression levels exhibited significantly worse scores across all quality-of-life domains (*p* < 0.001).

**Table 4:** Health-related quality of life data of patients studied across HADS-anxiety subgroups

Variable median(min.-max.)	Total (*n* = 192)	Normal (*n* = 47)	Borderline (*n* = 43)	Abnormal (*n* = 102)	*P*	H	η²	Effect size
Physical functioning	52.50 (0-100)	75 (0-100)	60 (0-100)	45 (0-100)	**0.0004**	11.167	0.048	Small to medium
Role limitations due to physical health	0 (0-100)	50 (0-100)	0 (0-100)	0 (0-100)	**< 0.001**	21.088	0.101	Medium
Role limitations due to emotional problems	0 (0-100)	100 (0-100)	0 (0-100)	0 (0-100)	**< 0.001**	34.508	0.172	Large
Energy/fatigue	45 (0–95)	50 (25–95)	50 (0–65)	35 (0–70)	**< 0.001**	38.310	0.192	Large
Emotional well-being	44 (0–92)	56 (36–92)	52 (12–72)	36 (0–80)	**< 0.001**	60.704	0.310	Large
Social functioning	50 (0-100)	75 (25–100)	50 (0-87.50)	50 (0-100)	**< 0.001**	26.055	0.127	Medium to large
Pain	45 (0-100)	77.50(22.50–100)	47.50 (0-87.50)	36.25 (0-100)	**< 0.001**	35.762	0.179	Large
General health	40 (0–75)	50 (20–75)	40 (10–60)	35 (0–75)	**< 0.001**	24.761	0.120	Medium to large

Bold: *p* < 0.05

**Table 5:** Health-related quality of life data of patients studied across HADS-depression subgroups

Variable median(min.-max.)	Total (*n* = 192)	Normal (*n* = 59)	Borderline (*n* = 59)	Abnormal (*n* = 74)	*P*	H	η²	Effect size
Physical functioning	52.50 (0-100)	75 (0-100)	55 (0-100)	45 (0-100)	**0.022**	7.635	0.030	Small
Role limitations due to physical health	0 (0-100)	50 (0-100)	0 (0-100)	0 (0-100)	**< 0.001**	21.867	0.105	Medium
Role limitations due to emotional problems	0 (0-100)	66.67 (0-100)	0 (0-100)	0 (0-100)	**< 0.001**	20.102	0.096	Medium
Energy/fatigue	45 (0–95)	50 (10–95)	45 (0–80)	35 (0–70)	**< 0.001**	30.500	0.151	**Large**
Emotional well-being	44 (0–92)	52 (16–92)	44 (4–76)	40 (0–72)	**< 0.001**	34.285	0.171	**Large**
Social functioning	50 (0-100)	62.50 (0-100)	50 (0-100)	43.75 (0-87.50)	**< 0.001**	25.549	0.125	Medium to large
Pain	45 (0-100)	67.50 (0-100)	55 (0-100)	45 (0-87.50)	**< 0.001**	20.013	0.095	Medium
General health	40 (0–75)	45 (5–75)	45 (5–70)	35 (0–60)	**< 0.001**	22.986	0.111	Medium

Bold: *p* < 0.05

## Discussion

Our study demonstrates a high prevalence of anxiety and depression among BD patients, that has a significant negative impact on their HRQoL. More than half of BD patients exhibited abnormal anxiety scores, and nearly 40% had abnormal depression scores. Gender differences were identified, with females experiencing higher anxiety rates. Severe disease activity, abdominal manifestations, and high ESR levels were strong key predictors of anxiety and depression. Our findings align with prior research on the psychological burden of BD. Numerous studies have established that BD patients exhibit elevated levels of psychological distress compared to healthy controls [5, 18, 19]. The erratic characteristics of BD, its relapsing course, and the occurrence of distressing and debilitating symptoms substantially elevate the anxiety and depression levels [20].

The quantitative findings of this study are enriched by qualitative data from the literature that illustrates the real experiences of BD patients. Patients with BD often characterize their experiences as an “exhausting phase,” characterized by a constant vigilance against potential disease exacerbations, uncertainty about the illness’s progression, and the burden of multiple manifestations [21]. The psychological distress quantified in our study, affecting over half of patients for anxiety and nearly 40% for depression, reflects these deeper experiential themes. Patients with visible manifestations such as oral or genital ulcers frequently report feelings of shame, social withdrawal, and stigmatization, which may compound the anxiety and depression [22]. In Arabic-speaking communities, where mental health stigma continues to impede the pursuit of psychological help [23], these lived experiences can present particularly significant difficulties. The high prevalence of psychological distress in the present study underscores the importance of creating supportive environments in which patients feel safe discussing their emotional struggles.

In comparison to previous studies, our research specifically highlights the influence of psychiatric symptoms and systemic disease severity on HRQoL outcomes. Similar findings have been reported in studies utilizing the HADS and SF-36 to assess BD-related psychological distress [10]. A recent study by Khabbazi et al. [24] highlighted that BD patients with higher disease activity reported significantly poorer SF-36 scores across all domains. Our findings also corroborate those of a review by El Hasbani et al. [6], which indicated that BD patients, especially those with neurological and mucocutaneous involvement, are at an increased susceptibility to anxiety and depression.

Our research adds to the growing body of literature by assessing Arabic-speaking BD patients, an understudied population. Regional variations in mental health outcomes and healthcare accessibility may influence psychological distress levels. Prior studies have suggested that stigma and limited mental health support in Middle Eastern populations may exacerbate psychological symptoms in BD patients [25, 26]. Furthermore, our study adds novel insights into the relationship between specific clinical manifestations and psychiatric distress in BD patients. Previous research has identified associations between BD and psychiatric disorders [27], and our multivariate regression analysis provides additional evidence linking high ESR levels, and abdominal symptoms, to anxiety and depression in BD patients. These results align with the results of previous research that highlights the relationship between systemic inflammation and neuropsychiatric disorders in systemic autoimmune diseases [28].

The increased frequency of anxiety and depression in BD may be explained by a number of reasons. First, a key factor in mood disorders is persistent inflammation, a characteristic of BD [29]. By affecting neurotransmitter function and neuroplasticity, pro-inflammatory cytokines like tumor necrosis factor-alpha (TNF-α), interleukin-1 (IL-1), and interleukin-6 (IL-6) have been linked to the pathophysiology of depression and anxiety [30]. These cytokines can impair serotonin metabolism, causing changes in mood regulation. Second, the unexpected nature of BD, which is marked by repeated painful ulcerations, ocular involvement, and neurological consequences, causes severe psychological anguish, raising the risk of mental health disorders [5]. Furthermore, the influence of BD on physical appearance, everyday activities, and social interactions exacerbates emotional distress, which is linked to anxiety and depression [31]. Finally, corticosteroid and immunosuppressive treatments used in BD management can have an impact on mental health, with corticosteroids being particularly linked to mood disorders [32].

Our regression analysis indicated a negative correlation between cyclosporine usage and anxiety symptoms. This can be clarified through biological and psychological aspects. Cyclosporine is a powerful calcineurin inhibitor that effectively suppresses T-cell activation and cytokine production, potentially reducing systemic inflammation linked to anxiety [33]. Patients exhibiting severe disease manifestations, such as refractory uveitis or vasculitis, who get cyclosporine may attain better disease management, leading to reduced symptom burden, fewer exacerbations, and a more favorable perceived prognosis [34], all of which could mitigate anxiety.

The current study illustrates a substantial inverse relationship between anxiety and depression scores and HRQoL in individuals with BD. The HADS showed that people with higher levels of anxiety and depression had lower HRQoL scores in every domain of the SF-36 Health Survey. These findings are consistent with prior studies that has emphasized the adverse effects of psychological distress on HRQoL in chronic inflammatory diseases [35, 36]. The regression analysis also verified that patients with elevated HADS-A and HADS-D scores demonstrated markedly poorer HRQoL outcomes (*p* < 0.001). Comparable findings have been documented in other chronic diseases, indicating that psychological distress exacerbates the perception of disease severity and impairs coping mechanisms [37].

In the current study, anxiety and depression contribute to reduced physical functioning and increased role limitations in BD patients. Our findings show that individuals with higher anxiety and depression scores report greater difficulties in performing daily activities. This is consistent with previous research that used the SF-36 in BD patients [5]. The interaction between psychological distress and physical symptoms, including fatigue, pain, and joint involvement, likely worsens physical disability. Moreover, inflammatory pathways implicated in BD, such as increased cytokine activity, may further contribute to both physical limitations and mood disorders [1].

The present study found that patients with anxiety and depression had much lower scores for emotional well-being. Individuals with mood disorders frequently experience increased disease-related distress and impaired coping mechanisms [38]. Additionally, role limitations due to emotional health were substantially higher in individuals with psychological distress, reflecting difficulties in maintaining occupational and personal responsibilities [39]. These findings align with existing literature on chronic disease populations, emphasizing the bidirectional relationship between psychological distress and disease burden [40] and the need for emotional support to improve drug adherence and disease outcome [41].

Fatigue is a common and debilitating symptom in BD, exacerbated by both psychological distress and inflammatory processes [42]. Our study found that patients with higher anxiety and depression scores reported lower energy levels and greater fatigue, corroborating findings from similar studies on HRQoL in chronic inflammatory diseases [43]. BD patients encountered a prolonged and intricate journey, regarded as an exhausting phase of their lives [44].The persistent nature of BD-related symptoms, coupled with mental health challenges, likely contributes to worsening fatigue. Moreover, pain perception is heightened in individuals with anxiety and depression, leading to greater functional impairment and reduced HRQoL [45].

Social functioning is significantly compromised in BD patients experiencing anxiety and depression. Our findings reveal that patients with psychological distress report greater social isolation, reduced participation in social activities, and increased interpersonal difficulties. Social stigma associated with visible BD manifestations, such as oral and genital ulcers, further exacerbates these challenges. Previous studies have shown that impaired social functioning negatively affects disease management and adherence to treatment [46], underscoring the importance of psychosocial support in BD care.

In the current study, BD patients with comorbid anxiety and depression perceive their general health more negatively compared to those without psychological distress. The diminished perception of overall health in BD patients may be related to both the unpredictability of BD flares and the chronic nature of the disease [20]. Furthermore, psychological distress has been associated with increased illness perception and symptom amplification, this contributes to poorer self-reported health status [47].

The findings of this study highlight the necessity of a multidisciplinary approach in managing BD, emphasizing the importance of integrating mental health care. Given the high prevalence of anxiety and depression among BD patients, a collaborative effort involving rheumatologists and psychiatrists is essential to ensure comprehensive patient care. This integration can enhance early recognition of psychiatric comorbidities, facilitating timely interventions. Furthermore, routine psychological screening using validated tools such as the HADS should be incorporated into BD management. The early detection of anxiety and depression can prompt the initiation of psychosocial support, behavioral therapy, and pharmacological interventions. Additionally, integrating patient-centered counseling and coping strategies into routine clinical practice may empower BD patients to better manage their condition.

This study offers several strengths that enhance its scientific rigor and clinical relevance. First, it provides a comprehensive assessment of the psychological burden of BD by examining the prevalence and impact of anxiety and depression on HRQoL in Arabic-speaking patients. The use of validated and culturally adapted tools, such as the HADS and the SF-36 Health Survey, ensures the reliability and validity of the findings. Additionally, the study employs a robust cross-sectional design with a relatively large sample size (192 patients), enhancing its statistical power. Furthermore, the study highlights key demographic and clinical predictors of psychological distress in BD patients, providing valuable insights for tailored multidisciplinary management strategies. Lastly, the findings emphasize the importance of integrating mental health care into BD management, offering a strong foundation for future research.

This study has several limitations that should be acknowledged. First, its cross-sectional design prevents the establishment of causal relationships between anxiety, depression, and HRQoL in BD patients. Longitudinal studies are needed to explore the temporal relationship between psychological distress and disease progression. Second, the dual recruitment strategy (hospital-based and online survey) may have introduced selection bias, as these subgroups may differ in disease severity, healthcare access, and socioeconomic status. Third, disease activity was assessed by patient self-report rather than validated clinical indices such as the BD Current Activity Form (BDCAF) or physician global assessment. Although self-reported disease activity has been used in prior studies and is closely linked to patient-perceived disease burden, the absence of objective validation limits direct comparison with clinician-assessed disease activity. The extensive univariate analyses presented in Tables 1 and 2 involved multiple comparisons, which may increase the risk of Type I errors. These analyses were primarily exploratory and descriptive, that intended to characterize the study sample and to identify candidate variables for subsequent multivariate regression modeling., we did not apply corrections for these comparisons, and results should be interpreted with this in mind. Another limitation is the underrepresentation of certain demographic groups, such as rural populations or individuals with severe disease who may be less likely to participate in online surveys. Furthermore, while validated Arabic versions of the HADS and the SF-36 were used, cultural differences in the perception of mental health symptoms may influence responses. Lastly, potential confounders such as medication side effects, socioeconomic stressors, and coping strategies were not comprehensively analyzed, which may affect the interpretation of psychological burden in BD patients.

Future research on the psychological burden of BD should focus on several key areas to enhance understanding and patient care. First, longitudinal studies are needed to assess the long-term impact of anxiety and depression on HRQoL in BD patients, identifying potential fluctuations in mental health status over time. Additionally, research should explore the effectiveness of targeted psychological interventions, such as cognitive-behavioral therapy (CBT) or mindfulness-based approaches, in alleviating psychological distress and improving overall well-being. Given the cultural and social factors influencing mental health perceptions in Arabic-speaking populations, further studies should investigate region-specific coping mechanisms, stigma, and healthcare-seeking behaviors. Expanding research to include neurobiological mechanisms linking inflammation and psychiatric symptoms in BD may also provide valuable insights into disease pathophysiology and novel treatment strategies. Finally, future studies should incorporate larger, multi-center cohorts with diverse demographic backgrounds to improve the generalizability of findings and support the development of comprehensive, multidisciplinary management plans that integrate psychological care into routine BD treatment.

This study highlights the significant psychological burden experienced by patients with BD, demonstrating a strong association between anxiety, depression, and impaired HRQoL. The findings indicate that a substantial proportion of BD patients experience clinically significant anxiety and depression, with disease severity, psychiatric symptoms, and inflammatory markers serving as key predictors of psychological distress. Furthermore, the presence of anxiety and depression was linked to poorer HRQoL outcomes across all measured domains, underscoring the need for a comprehensive approach that addresses both the physical and mental health aspects of BD management.

Given the substantial impact of psychological distress on disease perception and daily functioning, early screening and intervention for mental health disorders in BD patients should be prioritized. Multidisciplinary care models integrating psychological support with routine medical management may enhance patient outcomes and overall well-being. Additionally, culturally tailored interventions should be developed to address mental health stigma and improve access to psychiatric care, particularly in Arabic-speaking populations.

## Data Availability

The datasets used and/or analysed during the current study are available from the corresponding author on reasonable request.

## Abbreviations

ANOVA: Analysis of Variance
BD: Behçet’s disease
HADS: Hospital Anxiety and Depression Scale
HADS-A: Hospital Anxiety and Depression Scale- anxiety subscale
HADS-D: Hospital Anxiety and Depression Scale-depression subscale
HRQoL: Health-related quality of life
ISG: International Study Group
SF-36: Short Form 36
TNF-alpha: Tumor necrosis factor-alpha
